# Peer-led learning: a novel approach to promote rural healthcare interest among medical students

**DOI:** 10.3389/fmed.2025.1566472

**Published:** 2025-04-28

**Authors:** Grace Perez, Alyssa Groves, Lana Fehr, Aaron Johnston

**Affiliations:** ^1^Distributed Learning and Rural Initiatives, Cumming School of Medicine, University of Calgary, Calgary, AB, Canada; ^2^Cumming School of Medicine, University of Calgary, Calgary, AB, Canada; ^3^Arrowwood Medical Clinic, Vulcan, AB, Canada

**Keywords:** rural medical education, peer-led learning, peer-assisted learning, undergraduate medical education, distributed medical education, distributed learning, rural healthcare, community engagement

## Abstract

**Introduction:**

A persistent maldistribution of medical workforce exists across Canada, with rural areas facing a greater physician shortage. Medical education can be instrumental to increase physicians in rural communities, and medical schools have adapted strategies to generate interest in rural careers among medical students. Many of these efforts occur within formal structured curriculum. This study appraises the effectiveness of peer-led learning (PLL) as a novel approach in rural medical education to provide students with a better understanding of rural life and rural medical practice.

**Methods:**

This is mixed methods study using a survey and follow-up focus group discussion to evaluate a day-long educational experience organized and led by a medical student to their rural community. Quantitative data were summarized with descriptive statistics. Reflexive thematic analysis was conducted on qualitative insights to describe the students’ experiences and perceptions about the educational rural day.

**Results:**

Of 54 participants, 50 completed the survey and 13 consented for the follow-up focus group. Most (78%) were female, have non-rural origins (78%), with only 2 having Indigenous status. Majority (61%) have low familiarity with rural medicine. Trustworthiness scores for information about rural life and medical practice were higher for rural-origin peers and rural-origin faculty compared to other sources of information such as government websites, social media, and traditional media. Thematic analysis yielded three main themes: (i) informal teaching facilitated learning, (ii) trust in their peer enabled students to receive information more favorably, and (iii) students gained a better understanding of rural life and medical practice.

**Conclusion:**

This study demonstrated that medical students engage differently with peer-led learning activities about rural medical curriculum versus a formal teaching environment. Medical students are cautious about promotional information regarding rural medical education from formal sources but are less skeptical when learning from peers. Information about the way of life and healthcare needs in rural communities may be perceived as more credible and valid if coming from a peer, and hence, is more likely to be received favorably. Thus, when promoting rural education and careers, medical schools should work with rural-origin students, whose messaging may be considered more trustworthy than traditional sources.

## 1 Introduction

Globally, the medical workforce is suffering from a persistent geographic maldistribution with fewer doctors per head of population in rural communities compared to metropolitan areas (1, 2). Medical schools across Canada have made efforts to address the rural healthcare access crisis by working to generate interest in rural medicine among medical students (3–5). Many of these efforts occur within formal structured curriculum including clinical rotations, experiences, and clerkship placements in rural settings, inclusion of rural curriculum content in courses, and utilization of rural physicians as teachers and mentors (6, 7), as well as the recruitment of rural-origin students (8). Despite these efforts, medical student interest in Family Medicine rural careers continues to fall behind rural workforce needs. A recent report by the Canadian Resident Matching Service (CaRMS) revealed that the number of positions filled to Family Medicine residency training program was 85% for 2024 compared to 93% in 2014, marking the lowest first-round match rate in the past decade, which directly impacts the supply of family physicians for rural Canada (9). Notably, student recruitment from rural communities remains low and does not represent the Canadian population, with only 6.4% of medical students having a rural background compared to 18.7% of the population living in rural communities (10, 11). This may be partly due to the fact that medical schools use different policies and procedures to recruit rural and Indigenous students (2). Hence, most medical students are from urban locations and are unfamiliar with rural ways life and culture (12). Research has demonstrated that there is a difference in perceptions of rural life and rural medical practice depending on the student’s prior experience with rural communities (13, 14). Sadly, rural medicine is vastly misunderstood among students from urban centers, who often base their perceptions of rural medicine on stereotypes (12). Misconceptions and negative views about rural careers are a concern as these may dissuade medical students from participating in rural learning experiences such as rural rotations and, crucially, from eventual rural practice (15). In addition, feeling prepared for small-town living is an equally important predictor of physician retention in a rural community, as feeling prepared for rural practice (16).

This study explores peer-led learning (PLL) as a novel approach in rural medical education to provide medical students with a better understanding of rural life and rural medical practice. Outside of formal medical education curriculum, other conventional strategies such as student interest groups focused on rural medicine (17, 18), and exposure of students to quality rural experiences such as rural mentorship programs (19) have showed more promising results. Educational research has shown that techniques utilizing social learning experiences and involvement of peers are more effective than solitary learning approaches (20–22). Instead of a predefined curriculum, PLL is an informal approach where students learn with and from each other through interactions as peers or fellow learners (23, 24). PLL has been described as “the acquisition of knowledge and skill through active helping and supporting among status equals and matched companions,” where peers help each other learn and they themselves also learn in the process (25). Another study described the nature of PLL as “students learn a great deal by explaining ideas to others and by participating in activities in which they can learn from their peers” (26). Unlike structured curriculum, PLL is more adaptable, cooperative, and spontaneous. It utilizes various learning modalities including informal discussions, group activities, field excursions, and outdoor expeditions compared to more structured formats such as peer tutoring or task shadowing. The involvement of student peers in medical education has grown over time in many forms and contexts (25, 27), and its acceptability and advantages are well-established, including positive effects on academic performance, communication skills, and student satisfaction (28–34).

The authors of this study are from a department of a medical school in Western Canada, aiming to foster rural healthcare awareness and bolster interest by providing learners with educational opportunities and other rural experiences where students may gain an appreciation of rural life and learn more about rural practice. The goal is to encourage a robust pipeline of students who will consider future rural practice. Traditionally, learning experiences include the use of rural physicians as teachers and mentors, culturally immersive rural block rotations and longitudinal clinical rural clerkships. To better prepare medical learners, the authors seek innovative ways outside of conventional rural experiences to encourage students to consider things that traditional medical teaching may not. One such novel method used by the authors was adapting the living library concept as a learning modality to promote and facilitate interest in rural medical practice (15, 35). As another innovative approach, the authors have engaged with a student peer to help students gain fresh perspectives about rural life and rural medical practice. Under the guidance of a rural physician and member of faculty (LF), a medical student (AG) with a background in a rural farming community organized and led a day-long rural educational experience for their fellow students in their own community. The learning goals of the rural day experience were centered on understanding the lives of rural people and rural physicians with a view toward better caring for rural patients no matter what discipline or practice location the students eventually chose.

The objective of this research is to understand the impact on the learners of a rural day educational experience that is entirely peer-led and peer-organized. There is a paucity of research examining the impact of peer-led experiences on medical students’ attitudes toward rural life and rural medical practice. The authors hypothesized that medical students may engage differently with peer-led activities versus formal institutional activities and that trust in the information presented and ability to understand the environment from a peer perspective may be impactful.

## 2 Methods

### 2.1 Description of the rural day educational experience

The student peer (AG) organized a rural day-long educational excursion to their community of origin. The rural day was publicized to the entire first year medical class. Students signed up voluntarily for the rural day. The sign up and wait list was managed by the student leading the experience (AG). The organization of the day-long experience included a tour of a small-town hospital, meeting rural physicians and members of the community, and learning about the lives of farmers and what rural living looks like. Students were transported by bus from the medical school early in the morning. On their arrival, they broke into small groups for a tour of the community hospital and small group procedural skills stations (e.g., suturing, casting, intubation, IV starts, running codes, and laboratory skills), led by local physicians. After the small group sessions, the hospital’s clinical nurse educator ran an interactive code simulation with all the students. Following the clinical learning sessions, students were provided lunch and met members of the community, including some members of the peer’s friends and family. After the events at the hospital, the students travelled to the peer’s family farm where they were given a tour of the farm, taught about agriculture, introduced to the farm animals, and exposed to various agricultural technologies. The students then listened to a panel focused on understanding rural communities and the people who live in them, followed by a locally sourced dinner at the farm.

### 2.2 Evaluation of the rural day educational experience

This is mixed methods study using a sequential design approach without a comparison group to understand the potential impact of the rural day experience. For the quantitative portion of the study, an online post-event survey (Qualtrics 2020) was used, and for the qualitative phase, a follow-up focus group discussion was conducted with semi-structured questionnaire. The study received ethics approval from the University of Calgary Conjoint Health Research Ethics Board in May 2024.

#### 2.2.1 Post-event survey

After the rural day experience, the survey collected the participants’ demographic information (gender, nationality, age, rural origin, program of study, and year in the program and the students’ insights about the rural day experience). Data were also collected on students’ familiarity with rural medicine and the level of trust students placed on various sources of information about rural life and rural medical practice. To examine their attitudes toward rural life and medical practice, we asked the students to use 5-point Likert-type scales to rate their familiarity with rural medicine (*1 = not familiar at all to 5 = extremely familia*r) and their agreement to statements about aspects of rural medicine (*1 = strongly disagree, 2 = disagree, 3 = not sure, 4 = agree, and 5 = strongly agree*): as potential career option, scope of practice, rural life and work, interest in rural healthcare, and need for rural doctors. To evaluate the value of different sources of information about reality of rural life and rural medical practice, we asked students to rank the trust they put of information they get from rural-origin peers, rural-origin faculty, academic institution or medical school, official information provided by rural communities such as town websites, social media, and traditional news media. Finally, the following open-ended questions was presented: “*how were you impacted by this peer-led experience?*,” “*why was it important that this activity was peer-led?,”* and “*how do peer-led experiences factor into medical school?.”* At the end of the survey, students were asked whether they might be willing to join a focus group to further explore student perceptions and attitudes around their experience of the peer-led activity.

#### 2.2.2 Qualitative assessment

To explore the participants’ perceptions about their experience, qualitative insights were collected through a focus group discussion using a semi-structured questionnaire, as well as a qualitative open-ended questions in the survey. The semi-structured focus group guide is attached as Supplementary Appendix. An independent interviewer, with extensive experience in qualitative data collection and who was not part of the study team, facilitated the focus discussion via Zoom in May 2024. To ensure a conversational tone, the interview guide was applied flexibly. The focus group discussion lasted between 45 and 60 minutes. The responses were recorded through Zoom and transcribed verbatim by a professional transcription service, which removed any personally identifying information. For students who could not make it to the focus group discussion, they were invited to complete the semi-structured questionnaire on their own. There were five main questions as follows: (i) *“what made you participation in this rural day activity?,”* (ii) *“how did it mean to you that the activity was organized by a fellow student?,”* (iii) *“how would you describe the level of expertise shown by the fellow student for this activity?,”* (iv) *“what was the impact on you or what takeaway messages did you get from this experience?,”* and (v) *“how has this peer-led activity influenced your familiarity of the rural context?”*

### 2.3 Data processing and analysis

#### 2.3.1 Conceptual framework

**Figure 1:**
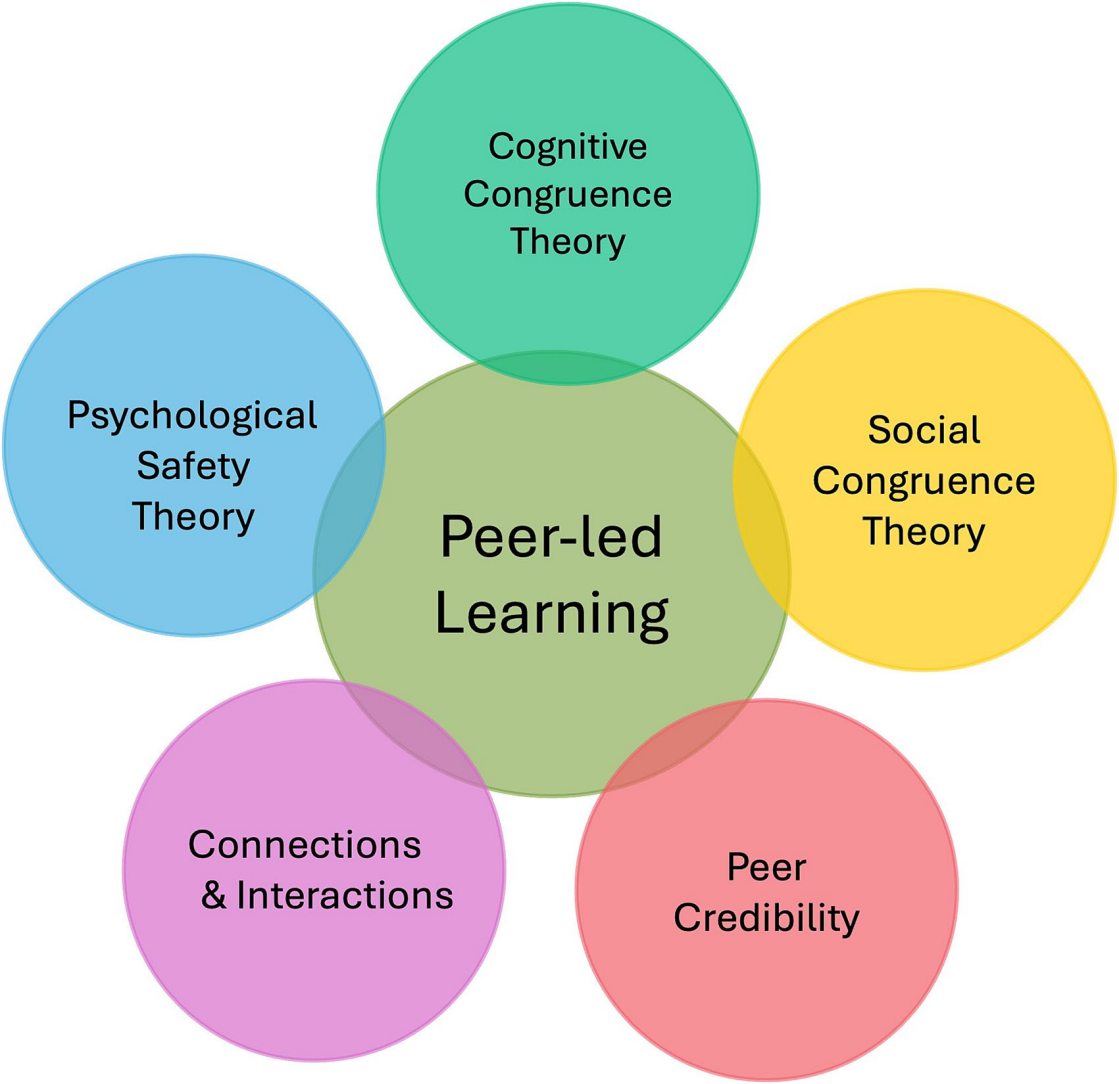
Schema of the key elements in the conceptual framework for peer-led learning (PLL).

The theories applied to peer-led learning vary extensively. To evaluate the rural day experience and to understand its potential impacts, the authors followed the conceptual framework suggested by other researchers of peer-assisted learning in medical education in our analysis (28, 29, 33, 36–39). Through this framework, depicted in Figure 1, the researchers posited that PLL happens when (i) *students and peer tutors share social congruence*, (ii) *students and peer tutors have comparable cognitive congruence*, (iii) *students feels a sense of psychological safety*, (iv) *peer tutors are considered to have credibility with the students,* and (v) *the connection and interactions in the group promote new learning*.

Social congruence implies same or similar socio-academic status between students and peer tutor, while cognitive congruence suggests that a relatively short knowledge distance exists between them (37). Psychological safety means feeling secure to speak up, voice concerns, or make mistakes without fear of repercussions (40).

#### 2.3.2 Quantitative data analysis

Quantitative data from the online survey were cleaned before further analysis using SPSS (version 29.0). Participants’ characteristic data were summarized using descriptive statistics. Attitude and perception scores were reported in terms of counts and percentages. Trustworthiness scores for the six sources of information for rural and rural medical practice were computed from the ranks provided by the students. The rank scores were reverse-coded using formula, *trustworthiness = max(rank) + 1 – actual(rank),* to reflect that an item ranked 1 would have the equivalent highest trustworthiness score of 6, while an item ranked 6 would have trustworthiness score of 1. Trustworthiness scores were also summarized using descriptive statistics and presented graphically as well.

#### 2.3.3 Qualitative data analysis

Audio recordings were transcribed verbatim by a professional transcriptionist and de-identified replacing names with pseudonyms during the coding process. Transcripts were analyzed using a reflexive thematic analysis approach, following similar steps used by Braun and Clark (41) and other qualitative researchers (42–44): (1) familiarization with the data, where authors (GP and AJ) read and re-read the responses to develop familiarity with what students are saying; (2) after familiarizing with the text, the authors separately identified meaningful words or phrases that form concepts or patterns (codes), (3) authors reviewed the codes together to define common themes, (4) authors discussed to reach consensus, with a third author adjudicating as needed (AG), and established a coding scheme, (5) the results were then recoded using this coding scheme, and (6) review of themes and selection of quotes to reflect the themes. Due to the relatively small number of transcripts, the coding was done manually and the study team met periodically to discuss emergent patterns.

### 2.4 Study team’s reflexivity and positionality

The research team consisted of two physicians (AJ and LF), a research staff (GP) with experience in mixed methods research and qualitative analysis, and medical student who is a novice researcher (AG). AJ is the associate dean for distributed teaching at the medical school, overseeing all distributed learning activities. LF is rural clerkship preceptor and works as a rural generalist physician from an Indigenous community. GP supports the various scholarship activities of the distributed teaching faculty, preceptors, and medical learners. AG grew up in a rural farming community. The authors were guided in their reflexivity by the work of Crabtree and Miller (45) and were mindful of their own biases and assumptions that they bring to this research. As a bias-mitigation measure, the research team used an independent interviewer to facilitate the focus group discussion.

## 3 Results

### 3.1 Study participants

Of the 54 students that participated in the rural day activity, 50 completed the post-event survey and their demographic description is provided in Table 1. Most were women (78%), aged less than 25 years (70%), and from non-rural origins (78%). Nearly half said they have domestic partners, but none have children. There were only seven participants that indicated being raised in a rural community and only two have Indigenous status.

**Table 1:** Demographic profile of student participants.

Demographic profile	*n*	%
Students who signed up for the activity	55	
Students who participated in the activity	54	
Students who provided post-activity data	50	100%
Gender		
Male	10	20.0%
Female	39	78.0%
Other	1	2.0%
Age group		
18–25 years	35	70.0%
26–30 years	10	20.0%
31–35 years	4	8.0%
36–40 years	1	2.0%
Ethnicity		
Caucasian/European/White	24	48.0%
Black/African	2	4.0%
Latin American/Hispanic (e.g., Mexican, Chilean)	2	4.0%
Middle Eastern/Arab/West Asian (e.g., Egyptian, Iranian)	1	2.0%
South Asian/East Indian (e.g., Pakistani, Sri Lankan)	8	16.0%
Asian (e.g., Chinese, Korean)	9	18.0%
Filipino/Pacific Islander	0	0.0%
Metis	1	2.0%
Mixed ethnicity	2	4.0%
No data	1	2.0%
Family status		
Single	26	52.0%
Partnered without children	23	46.0%
Partnered with children	0	0.0%
No data	1	2.0%
Rural or non-rural origin		
Rural	7	14.0%
Non-rural	39	78.0%
Not sure	3	6.0%
No data	1	2.0%
Indigenous status		
Yes	2	4.0%
No	47	94.0%
No data	1	2.0%
Career plans		
Family Physician	7	14.0%
Specialist Physician	27	54.0%
Not sure	15	30.0%
No data	1	2.0%
Specialist areas of interest		
Emergency Medicine	3	6.0%
Family Medicine	7	14.0%
General Surgery	8	16.0%
Geriatrics	3	6.0%
Internal Medicine	3	6.0%
Neurology	3	6.0%
Neurosurgery	3	6.0%
Orthopedics	2	4.0%
Obstetrics	1	2.0%
Pathology	1	2.0%
Pediatrics	1	2.0%
Psychiatry	1	2.0%
Radiology	1	2.0%

Thirteen students (26%) consented to be contacted for qualitative phase of the study. Of these 13 participants, 3 have rural origins while 10 have urban background. The authors planned to conduct separate focus groups for rural-origin and urban-origin students but could find a common time only for 7 participants from the urban-origin group for one focus group discussion. The 3 rural-origin students could not find a common time; however, 2 students still wished to participate and were provided the same questionnaire guide, used in the focus group, for completion on their own. In total, the qualitative data comprised of responses from multiple sources, i.e., 7 focus group participants, 2 questionnaire responses, and 50 text responses from the post-event survey. Across the multiple sources of data, the authors noted reaching a point of saturation as they encountered the same patterns and themes repeatedly during the analysis.

### 3.2 Quantitative data—descriptive statistics

Table 2 presents the students’ attitudes about rural medical practice and life. The authors considered “not sure” responses as non-favorable. Almost all students (94%) indicated disagreement that rural and urban communities have equitable access to healthcare, and almost all (96%) said they were interested in the health and wellbeing of rural communities. However, while 94% acknowledged the lifestyle benefits of living in rural communities and 98% said that the wide scope of practice would make for an interesting career path, only 45% indicated that rural medical practice would be a suitable career option for them. Despite this relatively low interest, it is encouraging that 76% thought it would matter to consider a rural practice and 92% would like to know more about the opportunities for rural medical practice.

**Table 2:** Attitudes and perceptions about rural medicine and the rural day experience.

Attitudes and perceptions	Strongly disagree	Disagree	Not sure	Agree	Strongly agree
Attitudes and perceptions toward rural medicine					
I’m interested in the health and wellbeing of rural communities	(0%)	(0%)	2 (4%)	20 (41%)	27 (55%)
Rural medical practice would be a suitable career option for me	1 (2%)	5 (10%)	21 (43%)	17 (35%)	5 (10%)
There are lifestyle benefits to practicing in a rural or remote community	1 (2%)	(0%)	2 (4%)	22 (45%)	24 (49%)
The wide scope of practice would be an interesting career path	(0%)	(0%)	1 (2%)	16 (33%)	32 (65%)
I am interested to know more about the opportunities for rural medical practice	(0%)	1 (2%)	3 (6%)	28 (57%)	17 (35%)
It would matter to consider practicing medicine in a rural community	1 (2%)	1 (2%)	10 (20%)	20 (41%)	17 (35%)
Rural and urban communities have equitable access to healthcare	14 (29%)	29 (59%)	3 (6%)	1 (2%)	2 (4%)
Rural day experience					
Peer-led events are an important way to learn about rural people and places	1 (2%)	0 (0%)	0 (0%)	9 (19%)	38 (79%)
Input from rural-origin students would improve the quality of rural curriculum and experiences in the medical school	1 (2%)	0 (0%)	3 (6%)	13 (27%)	31 (65%)

**Figure 2:**
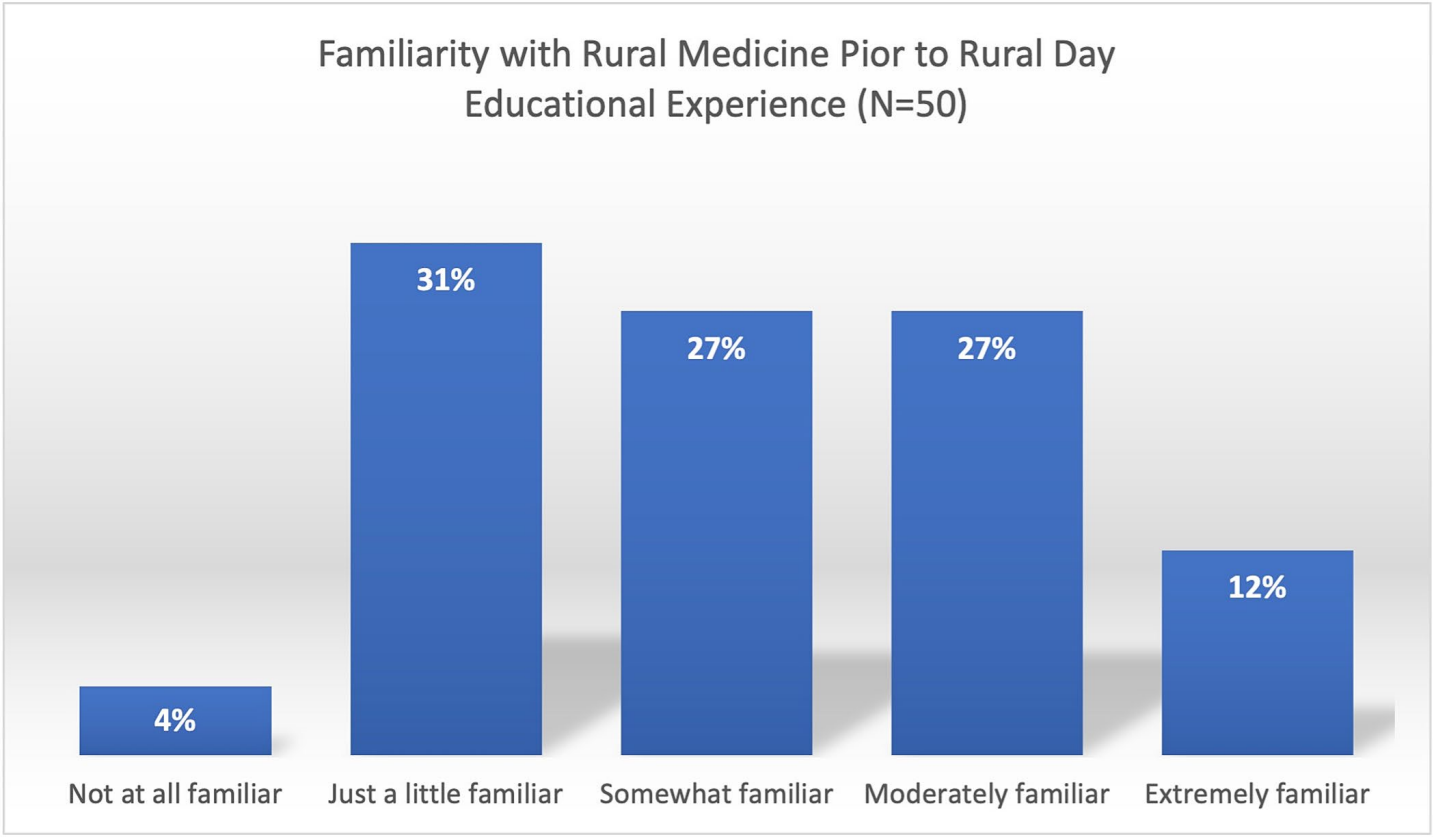
Familiarity with rural medicine prior to rural day educational experience, depicting that most students have low familiarity about rural medical practice.

Table 3 shows the proportion of students by the ranking of trustworthiness of the different sources of information for rural life and rural medical practice, while Table 4 describes the trustworthiness scores. Majority of the students have low familiarity with rural medicine, with only 39% indicating they were moderately to extremely familiar and the rest (61%) have little or no familiarity (Figure 2).

**Table 3:** Sources of information ranked by trustworthiness.

Trustworthiness of sources of information*	Ranking (*N* = 48)**					
	1	2	3	4	5	6
Source of information about rural life						
Rural-origin peers	36 (75%)	8 (17%)	3 (6%)	(0%)	1 (2%)	(0%)
Rural-origin faculty	7 (15%)	36 (75%)	4 (8%)	1 (2%)	(0%)	(0%)
Academic institution/medical school	1 (2%)	1 (2%)	9 (19%)	26 (54%)	9 (19%)	2 (4%)
Official information (e.g., town websites)	4 (8%)	2 (4%)	23 (48%)	14 (29%)	2 (4%)	3 (6%)
Social media (twitter, etc.)	(0%)	1 (2%)	8 (17%)	1 (2%)	11 (23%)	27 (56%)
Traditional media (news stories)	(0%)	(0%)	1 (2%)	6 (12%)	25 (52%)	16 (33%)
Source of information about rural medical practice						
Rural-origin peers	19 (40%)	13 (27%)	11 (23%)	4 (8%)	1 (2%)	(0%)
Rural-origin faculty	25 (52%)	21 (44%)	2 (4%)	(0%)	(0%)	(0%)
Academic institution/medical school	2 (4%)	14 (29%)	20 (42%)	8 (17%)	3 (6%)	1 (2%)
Official information (e.g., town websites)	2 (4%)	(0%)	10 (21%)	30 (62%)	4 (8%)	2 (4%)
Social media (twitter, etc.)	(0%)	(0%)	2 (4%)	4 (8%)	16 (33%)	26 (54%)
Traditional media (news stories)	(0%)	(0%)	3 (6%)	2 (4%)	24 (50%)	19 (40%)

* The sources of information were ranked from 1 to 6, with 1 being highest and 6 being the lowest. Rank scores were reverse-coded to reflect that an item ranked 1 would have highest trustworthiness score of 6, while an item ranked 6 would have lowest trustworthiness score of 1. ** Of 50 participants in the survey, only 48 provided responses to the trustworthiness questions.

**Table 4:** Trustworthiness scores of the sources of information.

Trustworthiness of sources of information*	N**	Min	Max	Mean	SD
Source of information about rural life					
Rural-origin peers	48	2	6	5.63	0.79
Rural-origin faculty	48	3	6	5.02	0.57
Academic institution/medical school	48	1	6	3.02	0.91
Official information (e.g., town websites)	48	1	6	3.65	1.14
Social media (twitter, etc.)	48	1	5	1.85	1.20
Traditional media (news stories)	48	1	4	1.83	0.72
Source of information about rural medical practice					
Rural-origin peers	48	2	6	4.94	1.08
Rural-origin faculty	48	4	6	5.48	0.58
Academic institution/medical school	48	1	6	4.02	1.04
Official information (e.g., town websites)	48	1	6	3.17	0.91
Social media (twitter, etc.)	48	1	4	1.63	0.82
Traditional media (news stories)	48	1	4	1.77	0.81

* The rank scores were reverse-coded to reflect that an item ranked 1 would have highest trustworthiness score of 6, while an item ranked 6 would have lowest trustworthiness score of 1. ** Of 50 participants in the survey, only 48 provided responses to the trustworthiness questions.

**Figure 3:**
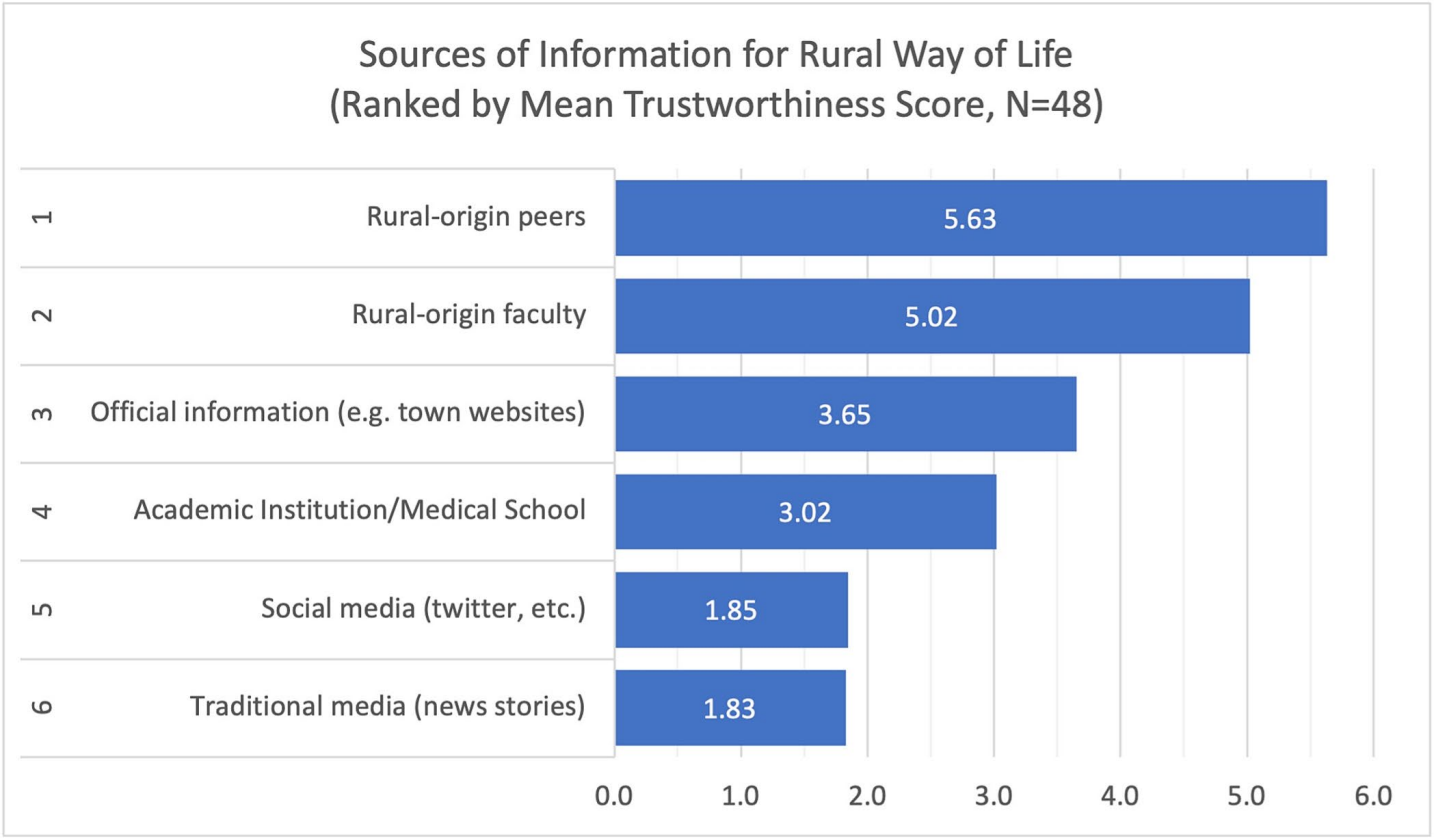
Trustworthiness scores of the sources of information about rural way of life, indicating high level of trust for rural-origin peers and rural-origin faculty.

**Figure 4:**
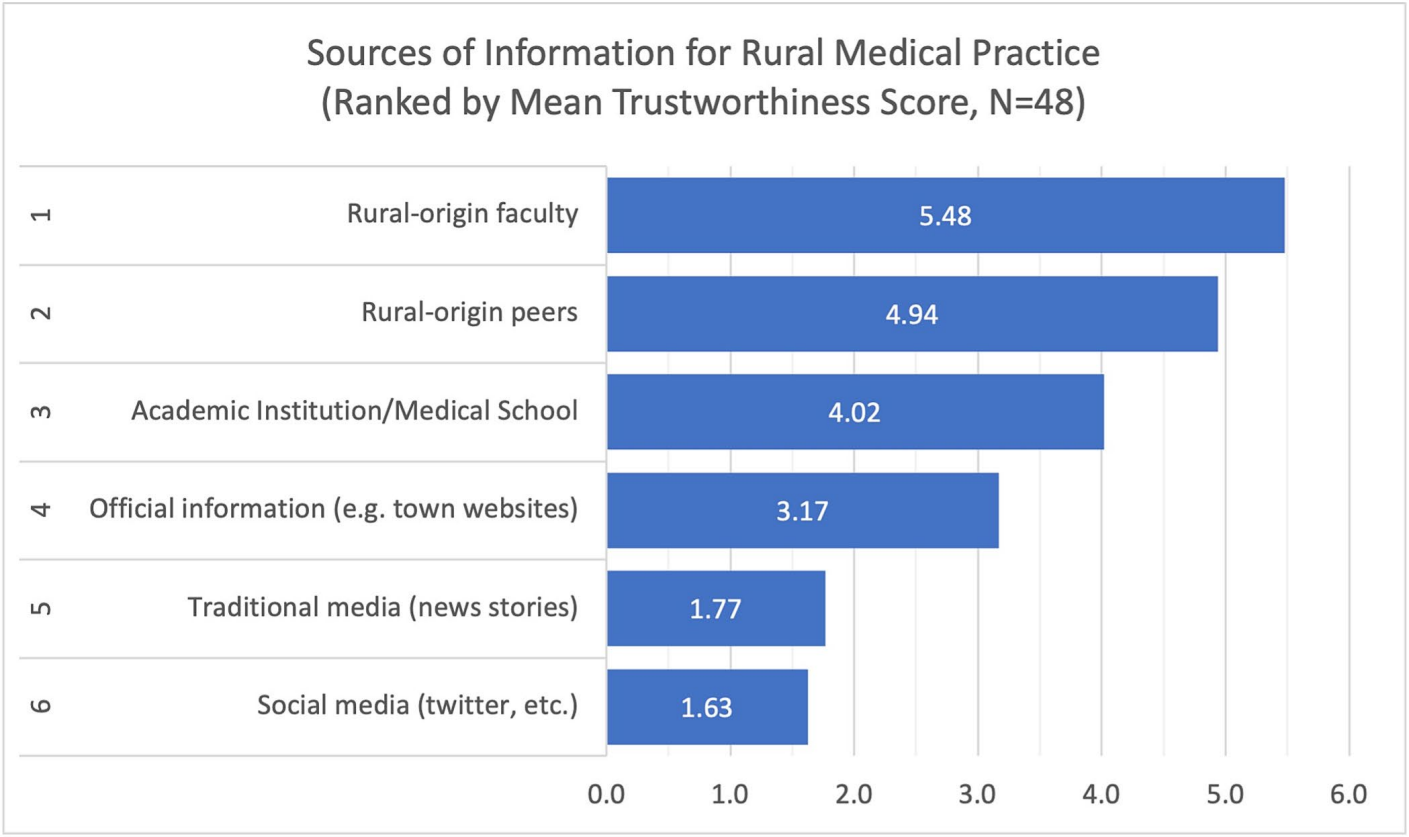
Trustworthiness scores of the sources of information about rural medical practice, indicating high level of trust for rural-origin peers and rural-origin faculty.

For information regarding rural life, students tended to trust most their rural-origin peers (mean = 5.63, SD = 0.79), rural-origin faculty (mean = 5.02, SD = 0.57), and official information from town websites (mean = 3.65, SD = 1.14) (Figure 3). For information about rural medical practice, students put more trust in rural-origin faculty (mean = 5.48, SD = 0.58), rural-origin peers (mean = 4.94, SD = 1.08), and the academic institution (mean = 4.02, SD = 1.04) (Figure 4).

### 3.3 Qualitative data—thematic analysis

Three main overarching themes emerged from the thematic analysis of textual data, namely, (i) the informal teaching facilitated learning, (ii) the trust in the student peer enabled students to receive information more favorably, and (iii) the rural day experience promoted a better understanding of rural life and medical practice. Relevant sub-themes are discussed under each main theme, with supporting verbatim responses provided as applicable. The verbatim responses are identified by participant number and the corresponding source of data, i.e., either focus group (FG), post-survey (PS), or questionnaire responses (QR). For example, “QR Participant 1” denotes student 1 who responded through the self-administered questionnaire.

#### 3.3.1 Theme 1: the informal teaching facilitated learning

The participants felt the rural day experience was engaging and facilitated learning. The sub-themes indicated by the transcripts for facilitation of learning included the (i) fun and relaxed environment, (ii) common knowledge base between learners and student peer, (iii) high expertise of the student peer about rural life, and (iv) enthusiasm and supportive motivation provided by the student peer.

i. The fun and relaxed environment of the rural day activity was a key element in learning.

A lot of the participants come from urban backgrounds, and the day trip to a rural town was regarded as refreshing and relaxing, as well as a unique learning opportunity.

- *I really liked that [the day] was divided into kind of two parts, that we had the morning at the hospital learning skills, kind of doing things that we do in class and all that. But going beyond that, too. Like, a lot of those skills we have not learned yet, or might not learn while we are in pre-clerkship, but then having the afternoon on the farm, more relaxed, where we are able to talk to classmates that maybe we do not usually talk to, or even AG’s family and friends. I really liked that separation because yes, kind of like someone else said, like, we are in class. We learn all these things and all that. So it’s nice to have that separation of a more social, while still learning about rural life. (FG Participant 6)*
- *It was really insightful to hear from health care professionals practicing in Vulcan, and also to hear from farmers who live in the area. I really enjoyed the opportunity to practice procedural skills, learn about rural life, and further connect with classmates. I also really enjoyed the animal therapy that came with meeting all the animals on the farm! (PS Participant 40)*

ii. The common knowledge level between learners and peer facilitated learning.

The participants noted that the learning was different because the student peer was their classmate and friend who knew what would benefit the students to learn.

- *[AG] knew what would be attractive to us, and that really came forward in what she decided to showcase in the day… Just the fact that we have such different backgrounds, bringing that in, whether it’s providing a different way to explain concepts or a different way of teaching the concepts, or “Maybe they did not touch on this part in lecture, but hey, I can provide this extra knowledge that might help you remember this better.” I think there’s definitely a lot of value in that. And kind of like someone else said, I cannot see medical school without peer interaction and peer expertise. (FG Participant 1)*
- *I think, we obviously spend a lot of time with AG and … she is a very well-organized person, so… she had put a lot of time and effort and it would be tailored to us. Like, I found the fact that she had, like, non-medical people on the panel, like, someone else to discuss, like, farmers and ranchers, like, that was very important. And she knew that that would something we’d find important because she knows us, and it was just, well – and I found that was, like, a personal touch that was different from just, like, doing procedural skills. (FG Participant 3)*

iii. The peer has high contextual expertise about rural life and served as ‘role model’ to fellow learners.

The peer’s rural background made the participants more receptive to their peer’s experience about living in a rural community.

- *She’s an expert in her lived experience, right? And a lot of it was so intimate to her upbringing [on the] farm. So I, you know, trust her, because this is, like, her reality, right? This is her life. So her, like, high expertise I guess. …. Like, just understanding people’s context, you know, where they come from, so where they are – you know, not that AG is representative of all rural people, right? But it’s interesting, like someone said, the perspective she brings in class which are very valuable. It’s amazing that we know a bit of the reason why she may see [those] or bring those, you know, perspectives on a medical issue or social issue that maybe someone else had not mentioned, you know. Someone who had the same upbringing would have thought. Like, seeing where somebody is raised and where they grew up. And then see how that pieces together. (FG Participant 5)*
- *The learnings of this experience could still work without being peer led, but having it peer led provided an unique lens in seeing the community through the individual’s eyes and in a way, made it more meaningful. It was the privilege of getting a into a major part of my peer’s life and gained an appreciation of these experiences that are new to me, are the daily life my peer and rural folks. (PS Participant 22)*

iv. The peer’s enthusiasm and supportive behavior motivated students to participate.

Being friends and classmates, the participants appreciated the student peer’s invitation and promotion of the rural day activity, which generated a positive response.

- *And she was just so happy about it, and so passionate about it, and that really rubs off. Like it’s very – not that it becomes sterile when it’s someone else doing it, but when it’s someone in their community, like, she was just grinning ear-to-ear the whole time, and it was very infectious. So that was very, very sweet and meaningful. (FG Participant 3)*
- *I think a big aspect of my desire to participate was seeing how excited and passionate AG was about this event! Seeing her joy at the thought of having so many of us come learn where she’s from and how great rural medicine can be really made me keen to participate. Along with this, getting a tour of a farm and meeting farm animals, plus getting to learn some new medical skills all made me super keen about the day! (QR Participant 1)*

#### 3.3.2 Theme 2: the trust in the student peer enabled students to receive information more favorably

The student transcripts also suggested that participants felt safe in the open and trusting environment. The absence of skepticism made participants free to be themselves which facilitated learning. The sub-themes included (i) trust in the peer and absence of perceived hidden agenda, (ii) non-judgmental space, (iii) existing interpersonal connection between students and peer, and (iv) student peer being a bridge between students and the community.

i. The students did not feel pressured about any hidden agenda.

The participants indicated that their peer did not make them feel as skeptical, in contrast to some information from the medical school, of selling the idea of practicing rurally, to the point it feels “being disproportionately shoved down their throats.”

- *It made me very open and trusting instantly, I did not feel as skeptical or guarded. Most of the information via the UME has felt like it was presented with rose colored glasses and like rural was being disproportionately shoved down my throat. I had initially come into medicine wanting to practice rural and after the UME propaganda block 1 was rebelling against it and really not keen for it. AG’s event felt very real, I felt like we were having very honest conversations with real people who were not trying to fool/convince us of one thing or another. The experience was truly just an experience where we could explore and ask genuine questions rather than feeling like an off putting recruitment event. (PS Participant 34)*
- *When someone you know and trust provides you with information, I think it makes it a lot easier to have full faith in what they have to say because you value their opinion and know that they are reliable. Institutions can definitely be reliable as well, but when a peer can share their lived experience with you, it makes it more personable (QR Participant 1).*

ii. The students valued the non-judgmental space.

The participants felt free to be themselves and express their perspectives without fear of ridicule.

- *But the one nice thing about it being a peer is that she understands that, and she understands the best ways to – like, you know, not “convince” people, but have people choose to live rurally and work rurally and spend time rurally is to show rather than tell. And I think that that really makes a big difference, because she understands, like – she understands the nuance in it too, right? Like, there are so many of us that will end up in urban centers for a variety of reasons, and she’s not going to shame or diminish that. Instead she wants to show that, you know, “OK, maybe you will not live there, but gee, why do not you locum?” (FG Participant 3)*
- *I think she’s also given us no reasons not to trust her, and all the reasons to trust her. (FG Participant 7)*

iii. The existing interpersonal connection between students and peer enhanced the learning.

The social connection to the student peer promoted personal growth and self-discovery among the participants.

- *I guess the big difference would be because we have so much in common with AG because we are students and we know her in that way. And we are also friends with her. So that already is different than learning about something from a professor from the school, there’s, like, a power imbalance, where there’s a lot of distance, like, understandably there needs to be professional distance, right? So that is already, I think, it flavors how we, you know, receive information. (FG Participant 5)*
- *Because it was peer-led, I felt like I could connect to the experience better, because I could step into the shoes of my peer and experience what their day-to-day life is like. I think as well, they had better insights on how to lead the day to make sure that it was smooth and insightful. (PS Participant 30)*

iv. The student peer served as a bridge between students and the community, facilitating effective knowledge transfer.

The student peer was a sociocultural ambassador for the community, and the participants felt welcomed into the community.

- *Having a peer lead this experience felt a lot more organic, like we were invited into the community and I also could see the pride the healthcare providers and community members had in being able to share with us, via our classmate. (PS Participant 27)*
- *But because they had a relationship with AG and we had a relationship with AG, that kind of bridged it. And then that was all part of it. That the community cares for each other because they know each other, and because then they knew why we were coming, that made it better for us. (FG Participant 6)*

#### 3.3.3 Theme 3: the rural day experience promoted a better understanding of rural life and medical practice among the students

The transcripts indicated that students gained a better appreciation of living in a rural community, the importance of relationships and getting to know people as “persons,” and how interesting a rural medical practice can be. The sub-themes included (i) appreciation of the community and rural life, (ii) appreciation of the community members as persons, and (iii) appreciation of what rural medical practice can look like.

i. The students understood how relationships and connectivity are integral to rural life.

- *It was so special to see AG’s community and how big a role they have in her life as well as the broader Vulcan community. The issues shared by the various members of the evening panel gave a broad view of the community from a healthcare lens but also from the lens of issues that are important to the community and how that intersects with health and wellbeing. Having a peer-led experience gave us a tangible tie into what rural life looks like. (PS Participant 16)*
- *Compared to other organized activities by the school, I think it was the relationship all of the people and town had with AG that made a big difference. The bonds formed between these individuals was very apparent and heartwarming to see … I think what made the biggest difference was how the town and AG really showed us how great being part of a close-knit community can be. (QR Participant 1)*

ii. The students gained a sense of the community members as “persons” by allowing them to put a name to a face.

- *As someone who grew up in an urban setting, this experience was amazing in terms of furthering the very little knowledge I had of rural living and rural medicine. I think to best treat patients whether in an urban or rural setting, it is important to understand their background and personal circumstances and even though I’ll never have the same experiences as someone who has grown up or spent a lot of time in a rural community, I hope I’ll be able to better understand these patients through some of what I learned. (PS Participant 41)*
- *I think for me, it really was the sense of community that I felt while at the rural day. Like, you say, “Oh, I met Such-and-such,” and they know who you are talking about. Whereas in an urban setting you say, “Oh, I’ve shadowed this doctor,” and they have no idea who that is. And so really just not only in terms of the hospital itself, but just the community too, and how many people showed up, and how many people knew each other was just really cool to see, and is definitely very different than what I’ve grown up with in an urban setting where you are just, like, another face, but to them you are actually a person. (FG Participant 1)*

iii. The students had the opportunity to see what rural medical practice can look like.

The participants had a chance to experience “rural” and helped their understanding of rural life and medical practice.

- *I think you brought up a good point with just, yes, hearing more about the work that they do, and how much land they have, and even the cost of some of the things they have to pay for, really just provides perspective so that if we see those patients in clinic it’s, like, “OK, well we know that it’s calving season. They need to get back to the farm to do that. So how do we manage that?” really just provides kind of that different perspective that we can then take into consideration when treating patients that are from a rural community, and do have a farm to go back to. (FG Participant 1)*
- *I also think it’s really nice to see the spectrum of rural medicine. … And I think it’s just very cool to see the breadth, and then also how you might be able to fit into a different site, because they are quite different. They offer different things, like, in terms of, like, how you can practice, where you can practice, how you can best [suit] the community and what your community looks like. I think that’s always real nice to see different things (FG Participant 7)*
- *Rural medicine can be extremely exciting and full of huge variety! Living/working within a small, closely bonded community appears to be a very positive experience. (QR Participant 1)*

## 4 Discussion

The training of medical students represents a continuously evolving field, with the involvement of peers as tutors increasingly adapted in medical education. In this study, a student peer organized and led fellow medical students for a rural day experience in their community of origin to meet with members of the rural community and rural medical practitioners. The objective of the research was to understand the potential impacts of this peer-led rural day experience among medical students. Most of the participants in this study were from urban areas with only 14% from a rural background and only approximately one-third of participants familiar with rural medical practice. This is consistent with literature that most Canadian medical students are from urban origin and thus would have low familiarity with rural life (11, 12). After the rural day experience, the findings showed that a high number of students were interested in the health and wellbeing of rural communities and indicated that it matters to encourage medical students to consider rural careers, with almost all expressing appreciation for the wide scope of practice involved in rural practice. While only 45% indicated they would consider a rural career, 92% would like to know more about the opportunities for rural medical practice.

This study also found that medical students have more trust in the information when presented from a peer perspective. The results indicated that the students regarded relevant information about rural life and living in a small town as most trustworthy when coming from rural-origin peers. For information about rural medical practice, the students put more trust in rural-origin faculty and rural-origin peers than their academic institution. This is consistent with the literature on social learning theory that peer tutors have high credibility and can influence learning (36).

Analysis of qualitative responses revealed three main themes. The first main theme was that the informal teaching facilitated learning. One sub-theme was the low level of formality of the peer-led rural day enabled a non-judgmental experience for the students and the students reveled in the fun and relaxed environment of the rural day. The lack of pressure normally associated with formal teaching can lead to increased comfort to ask questions, share ideas, and explore solutions to issues, which are fundamental aspects of the learning process. This non-formal environment allowed for concordance and balance of active involvement and enjoyment (28). A second prominent sub-theme was the common knowledge level and close educational distance between the learners facilitated learning as the student peer understood and presented information at the level where the students are. One student described that student peer knew what would be attractive to the students and worked around that on how to showcase the rural day experience; hence, this sub-theme aligns easily with the cognitive congruence theory of the conceptual framework (39). Closely related to the comparable knowledge sub-theme was that the peer has high contextual expertise about rural life and served as ‘role model’ to fellow learners (29). Another student observed that the student peer had the lived rural experience and understanding of rural context and this adds value to perspectives coming only from someone who would have the same rural upbringing. Similarly, these insights are consistent with Loda et al.’s cognitive congruence theory (39).

The second main theme was that the trust in the student peer enabled students to receive information more favorably. The foremost sub-theme was about how the students did not feel wary of any hidden agendas during the rural day experience. As one student stated, because they knew and trusted the peer, it made it a lot easier to have full faith in what the peer had to say and considered the information reliable. The learners also indicated that their peer did not make them feel as skeptical in contrast to the agenda-driven information from the medical school. Instead, the learners felt like they were just being given honest information needed to make an informed career choice. Another insight broached was that the experience felt like having very honest conversations with real people who were not trying to fool or convince students about rural careers so much so that it felt like the priorities hit the bullseye. These insights conform closely with the existing literature on social congruence theory that peer tutors are considered to have credibility with the students (36).

The second sub-theme was that the students felt safe in the open and non-judgmental environment. The lack of perceived judgment made the students feel less guarded and more secure to speak up and ask questions without fear of ridicule, especially for students who were unfamiliar with rural life and medical practice. Moreover, even students who were not considering a rural career were not shamed or made to feel awkward or defensive. These insights coincide fittingly with the psychological safety learning concept (40, 46).

The third sub-theme was the existing interpersonal connection between the learners and the peer contributed to the learning by eliminating professional distance. One student expressed that the absence of power imbalance flavored how information was received as students may have felt less intimidated. One student also said that because the rural day was peer-led, they felt like they connected to the experience better and that they could step into the shoes of their peer and experience what their day-to-day life is like. This finding is consistent with the importance of seeing the peer at the interpersonal level (38, 39), in that being of the same status or socio-academic standing, the learners felt comfortable with their peer and the rural experience to voice concerns and share opinions. This concept fits well with the social congruence theory of the conceptual learning framework (39).

The fourth and noteworthy sub-theme was that the student peer served as a bridge between participants and the community. The student peer’s relationship both with their community and with their fellow students facilitated effective knowledge transfer. Students noted that because it was peer-led, the experience felt more genuine and organic and like they were being invited, as opposed to being recruited, into the community and that made them feel immediately at ease. One student further elaborated that because of the relationship the student peer had with the community, the community gave full support and buy-in to the event and as such it made it so much better for the participants. These insights from this sub-theme affirm the learning concept that the connection and interactions in the group promote new learning (33).

The third main theme was that the peer-led rural day experience promoted a better understanding of rural life and medical practice. The first sub-theme was the appreciation and understanding of rural life. One student conveyed that having a peer-led experience gave them a tangible tie into what rural life looks like, what healthcare concerns the community face, and the challenges to the health and wellbeing of the community. Moreover, as the second sub-theme, the students not only gained an appreciation of what living in a small town looks like, but the experience allowed them to see community members as “persons.” With this understanding, the students were subsequently able to gain a sense of what a rural medical practice could look like, which was the third sub-theme. One student even expressed an appreciation of the wide scope of practice that rural doctors have and how rural practice can be an exciting and fulfilling career. These insights relate well to two learning concepts in the framework that peer tutors are considered to have credibility with the students (36) and that the connection and interactions in the group promote new learning (33).

The results of this study are justifiably consistent with the learning theories that make up the conceptual framework for peer-led learning. The peer-led rural day experience allowed for student reflection and students are often better able to reflect on and explore ideas when there are no teachers or institutional authority around to influence them (26). The students regarded the chance to learn from a peer to be important and a huge advantage. The triangulation of the quantitative results and qualitative insights provides convincing evidence that involvement of a student peer in a learning activity such as this rural day experience can effectively deliver the rural medical education curriculum. One student noted that their idea of rural medicine changed because of this rural day experience. The findings further support previous evidence that the involvement of student peers in medical education benefits learners, the academic institution, and even the peers themselves (29, 33, 34, 47).

### 4.1 Limitations

The sample size was limited by the passenger capacity of the chartered bus transport which could sit 55 passengers at most. The students who were acquainted well with the student peer may have been convinced to attend. Similarly, students who may have an inherent interest in rural medicine may have been persuaded to take part. There were more female than male students and more students who grew up in metropolitan areas than those raised rurally. No statistical comparisons between demographic groups were performed. Therefore, caution is needed in interpreting the results.

The study findings may have been influenced by some potential biases from the participants, such as recall bias, social desirability bias and the influence of peers during the focus group, and sponsor bias, which may have limited negative comments, despite the independent interviewer.

The sustainability of the peer-led model relies on the self-identification of rural learners interested in acting as champions. The Canadian rural context may not be applicable to other international settings. This version of peer-led information involves costs to the medical program which may not always be available. The types of peer-led activities and information sharing will necessarily vary depending on the peers involved, their own context, community of origin, and experiences.

### 4.2 What this study adds

This study reveals important findings about the involvement of peers in rural medical education that social learning experiences can help facilitate knowledge transfer, that students have higher trust in information coming from peers, and that peer-led experiences can help provide a better appreciation of rural life and medical practice. Medical training programs should consider how they can best present information on rural healthcare and rural careers to learners. Learners are cautious when this information comes from official sources, viewing it as agenda-driven. Input from rural-origin students could improve the quality of rural curriculum and experiences in the medical school.

Partnering with rural-origin medical students to provide information offers a potential pathway to deliver information to students in a more effective way. This study highlights the importance of peer-led information for current generation medical students. Medical schools can consider partnering with medical students with lived experience to best communicate around knowledge topics and issues that may otherwise appear agenda-driven. The importance of lived experience and novel ways of communicating with medical students about rural careers is consistent with other research by the our research group (35). While the context of this study was a specific peer-led rural experience, the findings of this study could be applied to a variety of challenging topics and could be a focus of further study.

## 5 Conclusion

This study demonstrated that medical students engage differently with peer-led activities versus a formal teaching environment, substantiating that PLL can be effectively utilized to deliver rural medical curriculum. Medical students are cautious about promotional information regarding rural medical education from formal sources but are less skeptical when learning from peers. Peer-led rural experiences are an effective way for medical students to learn about both rural medical careers and rural life. Information about the way of life and healthcare needs in rural communities may be perceived as more credible and valid if coming from a peer, and hence, is more likely to be received favorably. Thus, when promoting rural education and careers, medical schools should work with rural-origin students, whose messaging may be considered more trustworthy than traditional sources.

## Data Availability

The datasets presented in this study can be found in online repositories. The names of the repository/repositories and accession number(s) can be found below: The datasets generated and analyzed for this study can be found in the Open Science Framework repository, with URL https://DOI.org/10.17605/OSF.IO/W6ABE.
