# Supplementary material for: Peer-led learning: a novel approach to promote rural healthcare interest among medical students
Source: Front Med (Lausanne). 2025 Apr 28;12:1566472. doi: 10.3389/fmed.2025.1566472 (PMC12066776; doi:10.3389/fmed.2025.1566472)
Supplement: Supplementary file 1 [file Data_Sheet_1.PDF]

Appendix I

Peer Led Rural Experience Focus Group Guide

We are interested in hearing about your experience with the rural day activity. Your participation will create new knowledge that will guide programs and policy around the critical issue of the connection between medical education and healthcare delivery.

I. Reason for Participation

1. What made you participate in this rural experience activity?
   a. What was the main reason you participated?
   b. What other factors or aspects of the activity that contributed to your decision to participate?

II. Value of Peer-led Activity

2. How did it mean to you that the activity was organized by a peer or fellow student?
   a. Given that it was organized by a peer, what did you think of importance of the activity?
   b. Why was it important or unimportant that the experience was peer-led?
   c. What added-value did the activity have, as a peer-led endeavor?
   d. What did it feel like to explore an environment familiar to a peer and led by them?

III. Value of Peer-led Expertise

3. How would you describe the level of expertise shown in this peer-led activity?
   a. How would you describe the subject matter expertise of a peer or fellow student?
   b. What do think made this activity different from other extracurricular activities?
   c. How would you view information that comes from peers differently than the information from institutions? How so?

IV. Impact of Peer-led Experience

4. How did this experience impact you?
   a. What stood out in particular for you about this experience? Anything extraordinary or exceptional?
   b. What key takeaway messages did you get from this experience? Can you name a few?
   c. How do peer-led experiences and peer expertise factor into medical school?
   d. What do you think of peer expertise as part of the medical school experience?

V. Rural Familiarity

5. How would consider your familiarity with rural living? How so?
   a. How has this peer-led rural day influenced your familiarity the “rural” context?
